# Does Elimination of a Laboratory Sample Clotting Stage Requirement Reduce Overall Turnaround Times for Emergency Department Stat Biochemical Testing?

**DOI:** 10.7759/cureus.819

**Published:** 2016-10-06

**Authors:** Sarah Compeau, Michael Howlett, Stephanie Matchett, Jennifer Shea, Jacqueline Fraser, Rose McCloskey, Paul Atkinson

**Affiliations:** 1 Emergency Medicine, Horizon Health Network; 2 Emergency Medicine, Saint John Regional Hospital / Dalhousie University; 3 Family Medicine, Dalhousie Family Medicine, Saint John Regional Hospital; 4 Laboratory Medicine, Saint John Regional Hospital / Horizon Health Network; 5 Emergency Medicine, Saint John Regional Hospital; 6 Nursing, University of New Brunswick; 7 Emergency Medicine, Dalhousie University

**Keywords:** laboratory testing, turnaround times, length of stay, emergency department

## Abstract

Introduction: Laboratory turnaround times (TAT) influence length of stay for emergency department (ED) patients. We studied biochemistry TATs around the implementation of a plasma separating tube (PST) that omitted a 20-minute clotting step in processing when compared to the standard serum separating tubes (SST).

Methods: We compared laboratory TATs using PST vs SST in a prospective before-and-after study with a washout period. TATs for creatinine, urea, electrolytes, troponin, and N-terminal pro b-type natriuretic peptide (NT-proBNP), as well as hemolysis rates, were collected for all ED patients. Results were excluded if the TAT was four minutes or less (data entry error). We recorded the 90^th^ percentile response times (TAT90; the time for 90% of the tests to be completed). Statistical analysis used survival analyses, Mann-Whitney U tests, and Chi-square tests of independence.

Results: SST and PST groups were matched for days of the week, critical values, or hemolysis. There was a statistically significant reduction in median TAT and proportion completed by 60 minutes. However, the effect size was only two to four minutes in the In-Lab-TAT90 with the PST tubes for all tests, except B-type natriuretic peptide (BNP).

Conclusions: Reducing the machine processing time for stat blood work with PST tubes did not produce a clinically meaningful reduction of TAT. Clinically important improvement for Lab TAT requires process analysis and intervention that is inclusive of the entire system. Fractile response times at a 90^th^ percentile for TAT within 60 minutes may be an accurate benchmark for analysis.

## Introduction

Reducing the patient length of stay (LOS) in the emergency department (ED) is an important clinical and management goal. ED LOS is a reported quality indicator for the risk of morbidity and mortality, ED flow inefficiency, hospital crowding, and staff burnout [1-2]. Laboratory investigations are commonly performed on ED patients and are important across the spectrum of disease, from guiding the management of critical illness to improving time to patient disposition [3-4]. Since laboratory test turnaround time (Lab TAT) is an important component of LOS [5], Lab TAT is thus a quality indicator of laboratory service [6-8]. Delays in laboratory reporting may delay critical decisions about patient care, increase the length of stay, and decrease ED efficiency [4-5, 9].

Quality improvement processes, including Lean process improvements and Six Sigma initiatives, have been shown to improve ED LOS and Lab TAT [10]. The management team of the study examined patient LOS and found 60-70% of the 150 patient visits per day underwent laboratory testing. Although our Lab TAT goal for stat testing is 60 minutes, consistent with other labs in North America [8, 11-12], the audited 90^th^ percentile TAT (Lab TAT90; the time for 90% of laboratory tests to be complete) was consistently greater than 80 minutes. Laboratory TAT for ED patients was targeted for process improvement.

Laboratory TAT can be divided into pre-analytical (pre-lab specimen collection to arrival and in lab processing, e.g. centrifugation), analytical (total testing time on the instrument), and post-analytical times (time for the results to be posted to the ED information system) [13-15]. Our quality improvement process examined the pre-analytical stage of specimen handling since most time delays have previously been shown to occur at this stage [16]. Two collection vacutainers (specimen collection tubes) were available and validated for chemistry analysis in our hospital, providing an opportunity to test an intervention for the in lab processing time: BD Serum Separator Tubes (SST™) and Plasma Separator Tubes (PST™) (Becton, Dickinson & Co., Franklin Lakes, NJ) . Prior to the initiation of this study, the ED used SST™ for the collection of common chemistry tests. Use of SST™ requires a minimum of 20-30 minutes clotting time before the specimen can be centrifuged and subsequently processed for analysis [17]. Conversely, PST™ requires fewer in lab steps, as it does not require the sample to clot before being processed. These tubes contain lithium heparin as an anticoagulant to prevent clotting and a gel separator to allow for separation of the cell contents for analysis. Since PST™ can proceed to the analytical stage upon receipt in the laboratory, PST™ use should decrease the in lab processing time and, therefore, decrease total TAT [18]. To the best of our knowledge, there are no published studies demonstrating that use of PST™ vacutainers improves TAT compared to SST™ vacutainers in the ED setting. Our primary hypothesis was that using PST™ would reduce In-Lab-TAT of common chemistry tests, potentially improving ED efficiency, and a clinically meaningful reduction in total TAT [13, 15, 19-22]. We defined a clinically meaningful reduction in total TAT as 20 minutes.

## Materials and methods

We designed a prospective five-week before-and-after study, utilizing a planned practice change in the ED. The ED nursing staff drew all study laboratory specimens according to the agreed protocol. They used SST™ for two weeks, followed by a one-week wash-in period and a two-week PST™ intervention. The chemistry tests chosen for this study were: a) electrolytes, b) urea, c) creatinine, d) high sensitivity (hs) troponin-T, and e) proBNP. Estimates of sample size were made from historical laboratory volume data to provide 80% power at a significance level of 5%. Block randomization determined the study dates. All results were included during these time periods with the exception of tests from admitted patients boarded in the ED. TAT values < 15 mins for electrolytes, urea, creatinine, and < 25 mins for troponin and NT-proBNP were considered lab errors and excluded. Specimens were collected by trained ED nurses and sent to the onsite, nationally accredited hospital laboratory via a pneumatic tube system. Order entry, arrival in the lab, analyzer times, and time of report to the lab information system (Cerner Millennium 2011) (Cerner Corp., N. Kansas City, MO) were time stamped and electronically transferred to the ED information system (Allscripts Sunrise Clinical Manager v. 5.5, Emergency Care Module) (Allscripts Healthcare Solutions, Inc., Chicago, IL) to be viewed by the ED staff. To ensure no SST™ was used in the PST™ period, SST™ was removed from the ED carts during the intervention arm. 

All tests ordered STAT in our hospital during the study period were reviewed and cross-referenced to ensure all orders from ED were captured. Statistical analysis was performed using descriptive statistics in Microsoft Excel 2010

Ethical approval for the study was granted by the Research Ethics Board Horizon Health Network. Due to the nature of the study, we obtained approval to waive the requirement for informed consent from patients prior to sample collection.

The primary outcome was 90^th^ percentile test completion from specimen receipt in the lab to the result being reported (In-Lab-TAT90), a better indicator of laboratory performance than mean or median TAT [8, 11]: it captures much of the modifiable outlier effect and represents a standard of consistency required for ED care [23]. Survival analysis was used to determine the In-Lab-TAT90 and 95% confidence interval (CI) for each analyte. The proportion of tests completed in lab within 60 minutes was reported using Chi-square, and the distributions of each tube were also compared using median and interquartile range (IQR). The Mann-Whitney U test with 95% CI was also performed since our data was not normally distributed. Chi-square tests of independence were performed to assess differences with respect to day of the week, time of the day, critical values, and hemolysis. 

## Results

The study included 5,523 laboratory tests; 2,823 in the SST™ group, and 2,700 in the PST™ group (creatinine: 1,521, urea: 1,504, electrolytes: 1,573, troponin: 818, and NT-proBNP: 107).

**Figure 1:**
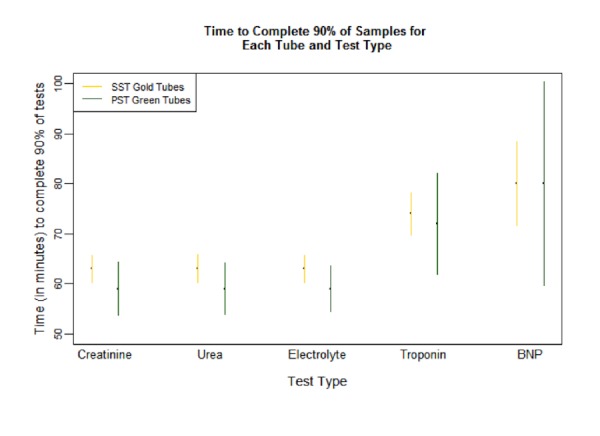
In-Lab-TAT90 Fractile Response Time, SST vs PST

Reductions for In-Lab-TAT90 with the PST™ tubes compared to SST™ did not reach statistical significance (Table 1, Figure 1). Effect sizes ranged from two to four minutes. The median In-Lab-TAT and the proportion of In-Lab-TAT completed by 60 minutes were statistically significant and higher for PST™ tubes for all tests, except NT-proBNP (Tables 2-3). The range of median difference for In-Lab-TAT was one to six minutes (Table 3).

**Table 1:** In-Lab-TAT90: Survival Estimates of 90th Percentile (Minutes) TAT = turn around time; NT-proBNP = N-terminal pro b-type natriuretic peptide

	Creatinine		Urea		Electrolytes		Troponin		NT-proBNP	
	N = 1,521		N = 1,504		N = 1,573		N = 818		N = 107	
	.90^%^	CI	.90^%^	CI	.90^%^	CI	.90^%^	CI	.90^%^	CI
SST Tubes	63	(60.3, 65.7)	63	(60.2, 65.8)	63	(60.3, 65.7)	74	(69.8, 78.2)	80	(71.7, 88.3)
PST Tubes	59	(53.7, 64.3)	59	(53.9, 64.1)	59	(54.4, 63.6)	72	(62.0, 82.0)	80	(59.7, 100.3)

**Table 2:** Proportion of Tests Completed in Lab Within 60 Minutes NT-proBNP = N-terminal pro b-type natriuretic peptide

	Creatinine		Urea		Electrolytes		Troponin		NT-proBNP	
	N = 1,521		N = 1,504		N = 1,573		N = 818		N = 107	
	N	Proportion (%)	N	Proportion (%)	N	Proportion (%)	N	Proportion (%)	N	Proportion (%)
SST Tubes	778	86.8	765	86.5	814	86.5	413	74.6	53	69.8
PST Tubes	743	90.0	739	90.4	759	90.6	405	83.7	54	64.8
P value	0.056		0.024		0.012		0.002		0.730	

**Table 3:** Differences in Median and IQR In-Lab-TAT (Minutes) IQR - interquartile range; TAT = turn around time; NT-proBNP = N-terminal pro b-type natriuretic peptide

	Creatinine		Urea		Electrolytes		Troponin		NT-proBNP	
	N = 1,521		N = 1,504		N = 1,573		N = 818		N = 107	
	Median	IQR	Median	IQR	Median	IQR	Median	IQR	Median	IQR
SST Tubes	38	(33, 49.8)	38	(32, 49)	39	(33, 49.8)	47	(64, 113)	52	(62, 98)
PST Tubes	34	(43.5, 70)	34	(43, 69)	35	(44, 71)	41	(54, 107)	51	(58.3, 89.8)
P value	< 0.001		< 0.001		< 0.001		< 0.001		0.953	

### Total TAT

**Figure 2:**
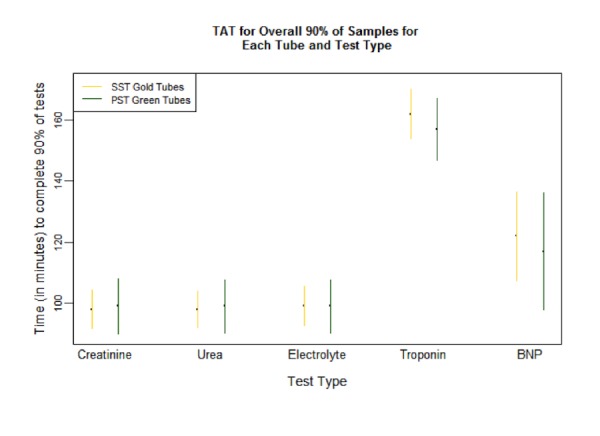
Total Lab TAT90 Fractile Response Time, SST vs PST

There was no significant reduction in TAT90 (Table 4, Figure 2). The median and proportion of total TAT completed within 60 minutes were significantly higher for PST™ tubes in all tests, except NT-proBNP, similar to In-Lab-TAT (Tables 5-6).

**Table 4:** Total Lab TAT90: Survival Estimates of 90th Percentile (Minutes) TAT = turn around time; BNP = B-type natriuretic peptide

	Creatinine		Urea		Electrolytes		Troponin		BNP	
	N = 1,521		N = 1,504		N = 1,573		N = 818		N = 107	
	.90^%^	CI	.90^%^	CI	.90^%^	CI	.90^%^	CI	.90^%^	CI
SST Tubes	98	(91.8, 104.2)	98	(92.0, 104.0)	99	(92.6, 105.4)	162	(154, 170)	122	(107.4, 136.6)
PST Tubes	99	(90.1, 107.9)	99	(90.2, 107.8)	99	(90.2, 107.8)	157	(146.8, 167.2)	117	(97.9, 136.1)

**Table 5:** Proportion of Tests Completed Total Lab Within 60 Minutes BNP = B-type natriuretic peptide

	Creatinine		Urea		Electrolytes		Troponin		BNP	
	N = 1,521		N = 1,504		N = 1,573		N = 818		N = 107	
	N	Proportion	N	Proportion	N	Proportion	N	Proportion	N	Proportion
SST Tubes	778	44.5	765	45.8	814	45.0	413	19.4	53	20.8
PST Tubes	743	60.6	739	61.6	759	59.2	405	37.0	54	31.5
P value	< 0.001		< 0.001		< 0.001		< 0.001		0.297	

**Table 6:** Differences in Median and IQR Total Lab TAT (minutes) IQR - interquartile range; TAT = turn around time; BNP = b-type natriuretic peptide

	Creatinine		Urea		Electrolytes		Troponin		BNP	
	N = 1,521		N = 1,504		N = 1,573		N = 818		N = 107	
	Median	IQR	Median	IQR	Median	IQR	Median	IQR	Median	IQR
SST Tubes	62	(50, 78)	62	(50, 76)	62	(50, 77)	80	(64, 113)	80	(62, 98)
PST Tubes	53	(43.5, 70)	52	(43, 69)	54	(44, 71)	70	(54, 107)	69	(58.3, 89.8)
P value	< 0.001		< 0.001		< 0.001		< 0.001		0.167	

### Confounding variables

There was no significant difference between SST™ and PST™ in the rate of critical values (4.4% vs 3.7%,  p = 0.222), weekend tests (26.4% vs. 27.7%, p = 0.3136) or hemolyzed specimens (6.8% vs. 6.0%, p = 0.2436). 

## Discussion

The total TAT results confirmed that the 60-minute goal for the processing of stat specimens from collection to reporting was not being met (Total Lab TAT90: 98-150 minutes, proportion TAT at 60 minutes 69.8 - 86.8%). In-Lab-TAT90 was 63 to 80 minutes. This confirmed that In-Lab-TAT was a reasonable target for quality improvement.

The implementation of new PST™ versus standard SST™ vacutainers did result in a statistically significant difference for median and proportion at 60 minute TATs. However, there was no significant difference for fractile in lab or total TAT90, with wide confidence intervals and small effect sizes. This was clearly not the 20 minute expected reduction in TAT, lacking true clinical significance for any single patient. The small PST™ effect size suggested that there were other important laboratory process issues beyond tube process time. Further investigation by the laboratory scientist confirmed large human factor process variability, as discussed below.

Volmar, et al. described stat test practices of 52 laboratories in 2013. For chemistry stat tests, the most common measured parameter was In-Lab-TAT (receipt of the specimen to the result report); 45 of the laboratories studied had established standards of median 45 minutes and TAT90 60 minutes. Reporting laboratories met expectations for the median standard 94.8% and TAT90 standard 99.0%. Compliance with benchmark In-Lab-TAT was monitored by 83.7% of participating laboratories. We demonstrated that a quality improvement initiative aimed solely at single factor improvement without considering other processes can fail, particularly where human process factors, such as workload, attention to bench process, or participation in the quality process, are not accounted for. Several studies have shown improvement using multifactorial process approaches; the type of methodology likely matters less than a multiple factor method that also engages those involved in the process [24].

Our data also demonstrate that the use of medians or means to review Lab TAT data gives an unreliable picture of lab efficiency. Wide confidence intervals may be more useful to show a pattern of unnecessary process variability. A fractile response time of 90% (TAT90) is a reasonable measure of reproducible system efficiency while recognizing there will be occasional outliers for valid reasons. Steindel demonstrated that a process specifically addressing outlier issues may be effective to improve TAT [21]. In our study, TAT90 was a more accurate measure of efficiency than either median or proportion, reflecting both effect size and magnitude of change needed to improve to the 60-minute benchmark.

Chien, et al. reported that a dedicated process control was able to significantly improve laboratory efficiency [23]. Our lab scientist proceeded to identify factors for improvement after reviewing the study results. Certain factors were identified that were likely to contribute to delays. Previously, all critical test results were required by policy to be repeated before the result was released, thus increasing TAT for these specimens. Tubes were often placed on a counter or in a specimen holder while lab staff attended to other duties, and processing was slowed by a limited staff complement at peak utilization times or during nights or holidays. Similar problems have previously been reported; Fernadez, et al. demonstrated that root causes for overall reporting time delay were lab attendant availability, recollection rate, the volume of tests for ED admitted patients, and order processing time [20].

The clinical implications of these long analytic TATs (in both SST™ and PST™ groups) mean that ED patients wait longer with less efficient patient care, delayed critical values, and lower clinician satisfaction. Multifactorial process review with staff and management engagement is important to understand interdepartmental lab-ED efficiency and meet benchmarks that enable clinically significant reductions in TAT. 

### Limitations

Total Lab TAT equals pre-lab, plus In-Lab-TAT. Although our data suggested that pre-lab times were also longer than expected, this was not a focus of our study. There may be factors in the pre-lab process that could improve total TAT. Areas of study may include the times for nursing staff to become aware of the new order, to draw the blood sample, and to prepare and send the sample in the pneumatic tube system.

Electrolytes, creatinine, and BUN results were very similar likely because the analyzer process and machine were similar, while troponin and NT-proBNP processes were different. There may have been analyzer processes to improve TAT that were not studied.

A collection of TAT data required collation of time information from more than one hospital database. While efforts were taken to ensure all tests were cross-referenced between systems, a unified data collection scheme would reduce the potential for data inconsistencies.

The effectiveness of the intervention may have been improved with more focused attention on specimen handling by the lab staff. It is important to note that, despite process change, failure to address such critical steps can result in reduced impact following interventions that make intuitive sense.

Although the SST study period had more tests conducted between 16:00 - 24:00 hours than the PST group (busier laboratory, less staff) and the PST had more tests between 00:00 - 08:00 hours (less busy laboratory, less staff), this did not increase the effect size. It is possible that In-Lab delay has more to do with inattention to detail rather than system problems, such as high volume of testing or staffing issues.

## Conclusions

Our laboratory required improvement in Lab TAT for stat chemistry tests to meet published benchmarks. We did not see the expected clinically meaningful improvement after eliminating a laboratory 20-minute clotting step with a new collection tube. Although we found a statistically significant difference in the in-laboratory median and proportion at 60 minutes lab TAT for PST versus SST tubes, this was not reflected at the 90^th^ percentile nor was the effect size clinically significant. The laboratory team proceeded to study the lab processes in more detail and successfully identified human factors in process control. Improvements of In-Lab-TAT for stat tests to the ED require a robust quality improvement methodology that is beyond single factor change and should use 90% fractile TAT as a benchmark rather than medians or means.

## Appendices

### Abbreviations

SST™ - Serum Separator Tubes 

PST™ - Plasma Separator Tubes 

TAT - Turnaround Time

IQR - Interquartile Range
